# Undercarboxylated Osteocalcin: A Promising Target for Early Diagnosis of Cardiovascular and Glycemic Disorders in Patients with Metabolic Syndrome: A Pilot Study

**DOI:** 10.3390/nu14142991

**Published:** 2022-07-21

**Authors:** Blanca Riquelme-Gallego, Laura García-Molina, Naomi Cano-Ibáñez, Francisco Andújar-Vera, Sheila González-Salvatierra, Cristina García-Fontana, Aurora Bueno-Cavanillas, Manuel Muñoz-Torres, Beatriz García-Fontana

**Affiliations:** 1 Department of Preventive Medicine and Public Health, University of Granada, 18016 Granada, Spain; briquel@ugr.es (B.R.-G.); lgarmol@ugr.es (L.G.-M.); ncaiba@ugr.es (N.C.-I.); abueno@ugr.es (A.B.-C.); 2 Instituto de Investigación Biosanitaria (ibs.GRANADA), 18014 Granada, Spain; sgsalvatierra@ugr.es; 3 Consortium for Biomedical Research in Epidemiology and Public Health (CIBERESP), 28029 Madrid, Spain; 4 Department of Computer Science and Artificial Intelligence, University of Granada, 18071 Granada, Spain; franciscoluisandujar@gmail.com; 5 Andalusian Research Institute in Data Science and Computational Intelligence (DaSCI Institute), 18014 Granada, Spain; 6 Department of Medicine, University of Granada, 18016 Granada, Spain; 7 CIBER de Fragilidad y Envejecimiento Saludable (CIBERFES), Instituto de Salud Carlos III, 28029 Madrid, Spain; 8 Endocrinology and Nutrition Unit, University Hospital Clínico San Cecilio, 18016 Granada, Spain

**Keywords:** metabolic syndrome, cardiovascular risk, diabetes, osteocalcin, Mediterranean diet

## Abstract

Lifestyle changes are causing an exponential increase in the prevalence of obesity and metabolic syndrome (MetS) worldwide. The most frequent complications of these are the development of diabetes (T2D) and cardiovascular disease (CVD). Accurate tools are needed to classify the cardiovascular risk (CVR) in the MetS population. In recent years, numerous biomarkers of bone metabolism have been associated with CVR. The aim of this study was to determine the levels of undercarboxylated osteocalcin (ucOC) in a cohort of patients with MetS and to analyse its association with MetS parameters and CVR as well as with T2D prevalence. A longitudinal study was conducted in which a MetS population was followed for one year. Weight change, adherence to the Mediterranean diet (MedDiet), ucOC levels, MetS parameters and CVR were analysed and CVR was calculated using different scores. Our results showed a decrease of CVR associated with a better adherence to the MetDiet resulting in higher HDL-C and ucOC levels though the improvement of MetS risk factors. This bone protein appeared as a potential biomarker to classify CVR in the MetS population, especially for MetS patients without prevalent T2D. Furthermore, ucOC serum levels could be good predictors of T2D prevalence.

## 1. Introduction

In recent years, several studies have reported a strong association between osteoporosis (OP) and cardiovascular disease [1,2]. An association between lipid profile disturbances and lower bone mineral density (BMD) has been described [3], as well as a key role of the angiogenesis process on bone repair [4] and local vascularization changes in bone disorders such as OP, rheumatoid arthritis, bone cancer or metastasis [5]. These findings support the connection of both pathological processes [6].

The coexistence of both pathologies could be partially explained by the unbalance in bone formation and resorption processes, which could be involved in vascular complications too [7] and by the presence of common risk factors such as menopause, obesity, alcohol intake, smoking, sedentary lifestyle, aging, inflammation, hyperglycemia and unhealthy diets. Healthy diets, such as the Mediterranean diet (MedDiet), characterised by a high intake of plant-based foods contribute to the increase and maintenance of BMD [8]. On one hand, although those dietary components produce alkalinity, specifically potassium and magnesium [9], on the other hand, high intakes of fiber can enhance calcium absorption and inhibit bone resorption by osteoclasts while maintaining the bone-forming activity of osteoblasts [10]. A MedDiet is characterised by a high intake of Monounsaturated fats (MUFA), which are associated with the maintenance of BMD and a lower incidence of fractures [11]. However, a high intake of saturated fats increases osteoclast survival and reduces intestinal calcium absorption, increasing its excretion in the urine. They are also associated with increased expression of inflammatory genes (*TNFα*, *IL-4*, *IL-17* and *P53*), leading to increased incidence of fracture [12]. Finally, the MedDiet targets adequate protein intake, which is necessary for the formation and maintenance of bone tissue, as well as stimulating the action of insulin-like growth factor 1, which in turn promotes bone growth and increases calcium absorption [13]. Classically, bone has been exclusively assigned a support and protector function; however, recent findings have shown the involvement of some bone proteins on homeostasis and energy metabolism regulation [14,15,16]. Osteocalcin (OC), a non-collagenous protein synthesized by osteoblast, has been associated with atherosclerotic disease parameters, such as pulse wave velocity (PWV) and intima-media thickness (IMT) in type 2 diabetes mellitus (T2D) patients [17] and in patients with prevalent atherosclerosis [18]. Nevertheless, its association with insulin secretion and insulin sensitivity has been the most remarkable disclosure [19]. The undercarboxylated fraction of OC is released into the bloodstream, acting directly on the pancreatic *β* cells and the adipocytes, improving glycemic homeostasis and increasing energy expenditure [20,21].

Metabolic syndrome (MetS) is a conjunction of central obesity and some cardiovascular risk (CVR) factors which lead to high socioeconomic costs globally every year [22,23,24]. MetS patients show 1.5 times more risk of all-cause mortality [25], 2 times more risk of cardiovascular mortality [26] and 5 times more risk of developing T2D than the healthy population [26,27]. However, large differences in cardiovascular risk among MetS patients have been described due to the great heterogeneity among its components [20]. On the other hand, MetS has been linked to the progression of other pathologies such as bone fragility and osteoporosis [28,29]. In this context, these patients set a good example of connection between atherosclerosis and bone demineralization processes. Despite the evidence that shows OC and undercarboxilated OC (ucOC) as energy metabolic regulators [30,31,32], and their association with individual CVR parameters in humans [33,34,35], the involvement of ucOC on global CVR in MetS patients remains unclear. 

The aim of this study was to analyze the usefulness of the serum ucOC levels as an indicator of global cardiovascular and T2D risk in a cohort of MetS patients in order to characterize properly these patients with a high heterogeneity to establish early preventive and therapeutic strategies for patients at higher risk.

## 2. Materials and Methods

A longitudinal study was conducted in 296 patients from primary care. All patients had obesity or overweight and were diagnosed with MetS according to the NCEP ATP III definition [36], meeting at least three of CVR criteria (high BP, impaired FPG, HDL-C and TG levels or increased WC). In order to ensure menopause status, men and women were aged older than 55 and 60 years, respectively, to make CVR comparable between sexes [37,38]. Outpatients were consecutively recruited from December 2014 to December 2016 in Granada (Spain) and followed up for one year. All of them were Caucasian and did not present active cancer or previous history of cancer, morbid obesity (≥40 kg/m²), any prevalent cardiovascular disease or bone diseases and/or any health problem that could interfere with the study protocol. The study was approved by the ethics committee of Granada in accordance with the principles of the World Medical Association’s Declaration of Helsinki. All patients signed the informed consent before being included. Anthropometric data were determined by standard procedures and sociodemographic variables were collected by questionnaire. Patients reported smoking status, pharmacological treatment and T2D prevalence. Systolic and diastolic BP (SBP and DBP) were obtained using a standard mercury sphygmomanometer reporting the mean BP by the equation (2 ∗ DBP + SBP)/3) [39]. Diet quality and physical activity were assessed at baseline and after 6 and 12 months of follow-up by the 14 items MedDiet adherence [40] and the Nurses’ Health Study for Spanish population [41] questionnaires, respectively. Blood samples were collected after an overnight fast and conventional analyses of lipid profile (total, HDL and LDL-Cholesterol and triglyceride (TG) level), fasting plasm glucose (FPG) and glycated hemoglobin (HbA1c) were performed. Serum ucOC levels were measured in duplicate by enzyme-linked immunosorbent assay (ELISA) according to Takara Bio, Japan instructions at baseline, 6 and 12 months of follow up. Intra- and inter-assay variations precision testing were consistent with reported by the manufacturer (6%–10% and 5.21%–8.33%, respectively). 

Although all MetS patients were treated as usual from primary care professionals, they were recommended to follow a healthy MedDiet to increase the consumption of olive oil, fruits, vegetables and nuts and decrease the consumption of meat, simple sugars and saturated fats and encouraged to practice physical activity according to the WHO recommendations (at least 150–300 min of moderate-intensity aerobic physical activity; or at least 75–150 min of vigorous-intensity aerobic physical activity; or an equivalent combination of moderate- and vigorous-intensity activity throughout the week) [42]. 

In order to classify CVR in MetS patients, a Z-score (CV-ZS) was calculated at baseline and during follow-up using the NCEP ATP III CVR criteria [36]. The mean-centering and standard deviation (SD) normalization were sex-specific for each variable by the (x-mean (x))/SD equation. Global CVR punctuation was the average of the Z-scores of mean BP, TG levels, WC, FPG and the inverse Z-score of HDL-C [43]. The CVR Framingham score was estimated by the Wilson P.W. et al. equation including age, T2D prevalence, smoking and sex-adjusted CVR variables (total cholesterol, HDL-C, LDL-C, systolic and diastolic BP) [44]. The cut-off point corresponding to the 25th percentile of baseline log ucOC levels was used to evaluate the cardiometabolic profile and CVR of MetS patients at baseline and at 6 and 12 months follow-up.

Statistical analysis was performed using SPSS version 22.0 software (SPSS, Inc., Chicago, IL, USA). The normality of the variables was checked by Kolmogorov-Smirnov test and a log transformation was performed for serum ucOC levels. Continuous variables with normal distribution were presented as mean ± SD, and categorical variables were expressed as percentages. Student’s t and ANOVA tests for continuous variables and χ^2^ test for categorical variables were used to determine statistical differences among the means of two or more groups. ANCOVA model was performed when an adjustment by covariates was required. The Pearson’s correlation coefficient was used to analyze the associations between continuous variables. The variables influencing CVR such as age, sex, smoking, sedentary activities and pharmacologic treatment for MetS (lipid-lowering, antidiabetic and antihypertensive drugs) were identified by multiple linear regression analysis. Multiple logistic regression model was applied to analyze ucOC as an estimator of T2D. A ROC curve was performed to estimate the usefulness of circulating ucOC levels as a marker of T2D risk. Values of the area under the curve (AUC) higher than 0.75 indicate a good predictive performance [45]. Statistical significance was set at *p* < 0.10 for multiple linear regression analysis and at *p* < 0.05 (two tailed) for the other analyses.

## 3. Results

Baseline characteristics of the study population according to sex are shown in Table 1. The proportion of women and men was 21.7% and 20.0%, respectively, of patients that had been previously diagnosed with T2D considering the American Diabetes Association criteria [46], with no differences by sex. More than 90% of the patients were hypertensive, 72.6% were obese and 94% of the females and 87.4% of the males showed high risk waist circumference (WC). The prevalence of dyslipidemia was similar for both sexes, however men showed significantly lower baseline HDL-C levels and women showed significantly higher LDL-C levels. Regarding lifestyle habits, mean MedDiet index was 8.6, without significant differences by gender, and men showed a higher prevalence of smoking and sedentary habits. In addition, males showed significantly lower ucOC serum levels and higher CVR measured by CV-ZS and Framingham score. 

**Figure 1:**
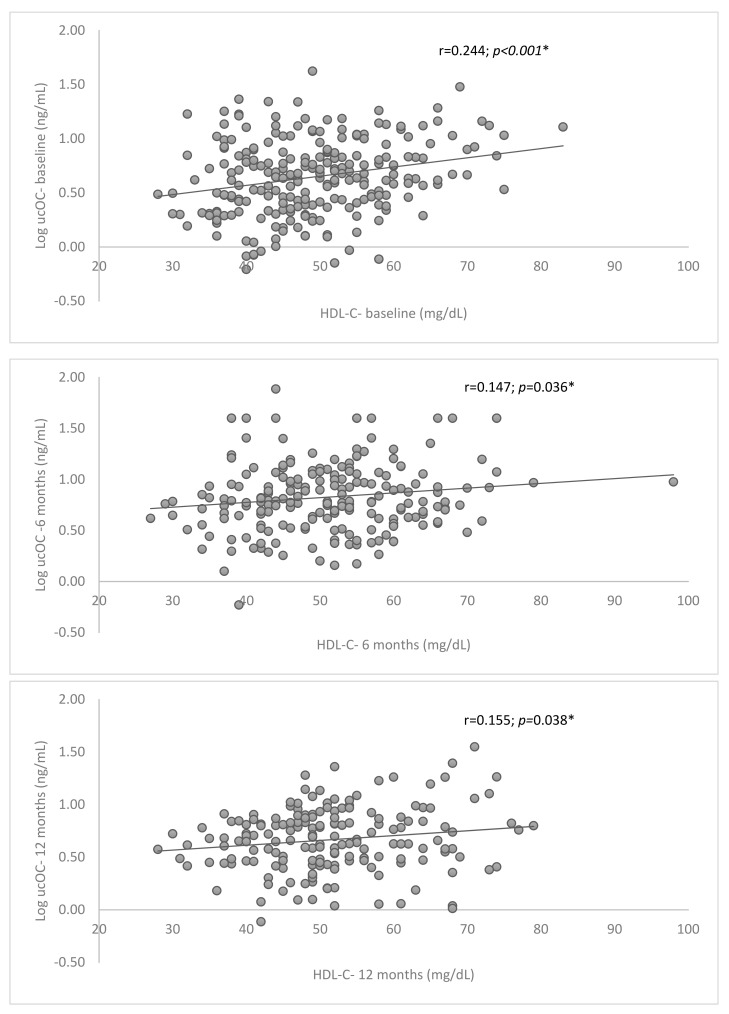
Relationship between serum ucOC levels and HCL−C at baseline, at 6 and 12 month follow-up. * Spearman correlation test < 0.05.

Table 2 shows the evolution of MetS risk factors (anthropometric measures, lipid and glycemic profile), MedDiet index, serum ucOC levels and CVR scores at the 6 and 12 month follow-up. The general population showed a general improvement especially at the 6 month follow-up. BMI, WC, HDL-C and BP decreased significantly, which slightly reduced total CVR measured by CV-ZS and Framingham scores. Mean MedDiet index increased above the recommended (nine points) and lipid and glycemic profiles showed an improvement, but only serum TG showed significative differences during the follow-up period. Serum HDL-C and ucOC levels increased significantly at the 6-month point but decreased again at the 12-month point, showing a significant correlation during the follow-up (Figure 1). 

Table 3 shows the variables correlated with serum ucOC levels. Apart from HDL-C levels, ucOC showed a significant negative correlation with HbA1c levels and CVR at baseline measured according to CV-ZS and Framingham, as well as with sex.

Serum ucOC levels increased from baseline 6.06 ng/mL to 9.44 ng/mL at 6 months and decreased to 6.39 ng/mL at the 12-months follow-up. We compared ucOC levels between patients with and without prevalentT2D at baseline, at the 6 and 12 month follow-up. Since ucOC levels were correlated with sex, we adjusted for sex and age (Table 4).

The independent association of ucOC serum levels (log) and CV-ZS was estimated through multiple linear regression model adjusting for age, sex, smoking, sedentary status, prevalent T2D and any MetS pharmacological treatment previously described. Baseline serum levels of ucOC appeared as an independent variable associated with CV-ZS for non-T2DM patients (Table 5). However, this association was not statistically significant at the 6- and 12-month follow-up analysis. 

In order to analyze the influence of serum ucOC levels on CVR factors, they were compared according to the 25th percentile of the logarithm of baseline ucOC serum levels (0.92 ng/mL) previously reported [43] by ANCOVA analysis, adjusting for sex (Table 6). This cut-off point was applied at the 6- and 12-month follow-up. A cardiometabolic profile were significantly disturbed when ucOC levels were below 0.92 ng/mL, and thus CVR estimated by CV-ZS was higher for these patients during the follow up period.

Finally, the relationship between serum ucOC levels and T2D prevalence was analyzed by performing logistic regression including biological variables classically linked to T2D (age, sex, sedentary status, lipid profile and WC) in addition to ucOC serum levels as independent variables, showing a negative association (OR = 0.051; [0.011/0.226], *p* < 0.001). 

**Figure 2:**
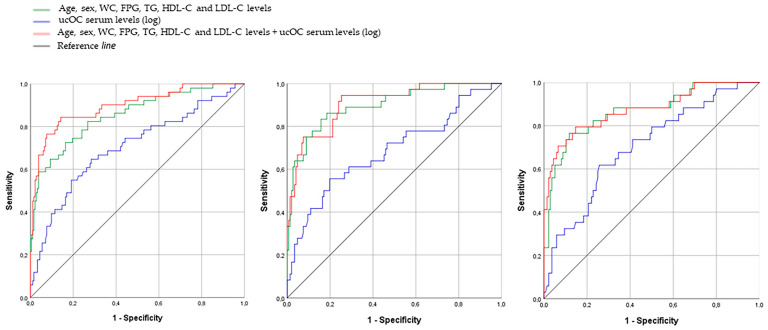
ROC curve to analyze the utility of ucOC serum levels (log) as an indicatorof T2D prevalencein MetS patients at baseline and after the 6- and 12-month follow up. ucOC: undercarboxylated osteocalcin. FPG: fasting plasma glucose; HDL: high-density lipoprotein; LDL: low-density lipoprotein; ucOC: undercarboxylated osteocalcin.

An ROC curve was performed to analyze circulating ucOC ability to identify T2D prevalence. The model including ucOC serum levels best described T2D prevalence, since it showed the highest area under the curve (AUC = 0.894; *p* < 0.001). This trend remained at the 6- and 12-months follow-up (OR = 0.98; [0.96/0.99], *p* = 0.005 and (OR = 0.11; [0.01/0.70], *p* = 0.019 respectively) (Figure 2). 

## 4. Discussion

Our results showed a decrease of CVR associated with a better adherence to the MetDiet resulting in higher HDL-C and ucOC levels in MetS patients. SerumucOC showed an association with HDL-C levels after one year of follow-up and seems to be a potential biomarker to classify CVR in the MetS population, especially for MetS patients without prevalent T2D. Finally, circulating ucOC appears to be a good predictor for the risk of developing T2D in MetS patients. Patients with ucOC levels below the 25th percentile showed six times more of possibility of suffering T2D and these results were confirmed at the 6- and 12-month follow-up. The reversion of MetS factors and the reduction of CVR through lifestyle habit improvement have been largely documented [38,39]. In this cohort of MetS patients an increase of mean MedDiet adherence score and a slight reduction of sedentary activities is observed. The consumption of olive oil and nuts is associated with the increase of HDL-C levels which correlates with ucOC levels during the follow-up, as previously reported in some studies [47,48,49].

Our results point to ucOC as a potential biomarker capable of stratifying CVR in MetS patients. In this way, our results show a strong association between cardiovascular risk factors (CVRFs) and circulating ucOC and observing significative lower levels of ucOC in those MetS patients presenting higher CVR. Considering the close association of serum ucOC levels with CVRFs, we evaluated its role as a predictor of CVR in our study population. Our results showed that serum ucOC level is an independent estimator of CVR adjusting for lifestyle variables and pharmacologic treatment at baseline. This association was stronger when patients with prevalent T2D were excluded from the analysis since glucose levels are the main regulators on energy [50,51,52,53] and the presence of diabetes is the main risk factor for CVD rather than circulating ucOC. Agreeing with our results, other studies have previously reported the association between the individual CVR factors and total OC and ucOC [48,54,55]; however, this is the first study that shows an association with a total score of cardiovascular risk based on the MetS factors.

In order to validate the cut-off point established in our retrospective study as a threshold for stratifying CVR [43], the cardiometabolic profile of the study patients was analyzed during the follow-up period according to the cut-off point of 2.53 ng/mL ucOC. Our results showed an association between lower ucOC levels and worse cardiometabolic profile and, thus, with higher CVR in terms of CV-ZS score during the whole follow-up period. Similar results were reported in a cohort of elderly men, showing a positive correlation between higher ucOC/OC ratio and lower incidence of myocardial infarction [56].

The better results in terms of improvement of CVR associated with higher HDL-C and lower ucOC levels were found at the 6-month follow-up when the participants were more aware of the intervention program. However, the strength of ucOC as a predictor variable of CVR was weaker at this point since the proportion of patients with values of circulating ucOC lower than 2.53 ng/mL was very low due to the improvement of lifestyle habits. Nevertheless, a relaxation in lifestyle habits was observed after 12 months of follow-up, thus the number of patients with values of ucOC below 2.53 ng/mL was raised again and the predictive value of serum ucOC for CVR was nearly significant. 

Based on the close association between ucOC and CVRFs, recent studies carried out in animal models have reported its effectiveness in vitro as a therapeutic treatment on atherosclerosis, showing an increase on capillary density and neovascularization and improving myocardial fibrosis [57,58].

Finally, the assessment of the usefulness of circulating uCOC for prediction of T2D risk in MetS patients positioned the ucOC as a powerful predictive strategy. Since MetS patients show an important heterogeneity, some of them may suffer higher glycemic disturbance, thus they will be at higher risk of T2D. Our results showed that patients with baseline levels below the cut-point of 2.53 ng/mL showed six times more of a risk of T2D. These results were confirmed at the 6- and 12-month follow-up, agreeing with previous findings [59]. These findings suggest that the measurement of serum ucOC levels could represent a promising screening tool in order to classify MetS individuals at high risk of developing T2D.

However, this study has some limitations. On the one hand, it is an observational study, so there is a possible residual confounding in the results due to a classification bias introduced by the self-reported data. On the other hand, because it is a population with CVR, it could lead to a social desirability bias, increasing the score on the lifestyle scales. In addition, total OC, N-MID OC, vitamin D and K, which may have an influence on serum ucOC values, were not determined. However, it should be noted that the main strength of this study is the novel assessment of CVR in patients with MetS using a global score of the accepted variables for the diagnosis of MetS, which allows a more accurate characterization of CVR in this heterogeneous population. Additionally, in contrast to other studies, confounding factors have been considered such as the presence of T2D and the pharmacological treatment for the management of MetS. This is therefore the first study to show the relationship between serum ucOC levels and the global CVR in this population. Furthermore, its considerable sample size and therefore its statistical power provides robustness to our results. Another strength is the longitudinal design, as the 12-month follow-up allows us to establish a causal relationship between weight loss due to the improvement of lifestyle and the decrease of CVR in MetS patients.

In conclusion, at the one year follow-up, it was shown that the improvement of lifestyle in a sample of MetS patients may have a double effect on their cardiometabolic health: on one hand through anthropometric changes and the improvement of MetS parameters, and on the other hand, by the increase of ucOC levels, which improves glucose homeostasis and insulin sensitivity by its action on beta pancreatic cells. Thus, ucOC is postulated as a good biomarker to classify T2D and cardiometabolic risk in such a heterogeneous population as MetS patients and to initiate early intervention in those at higher risk.

## Figures and Tables

**Table 1:** Baseline characteristics of the study population by sex.

	Males (N = 135)		Females (N = 161)		*p*
T2D (%)	20.0%		21.7%		0.697
>7 h of sedentary activity (%)	57.0%		36.0%		<0.001
Smoking (%)	16.3%		8.1%		0.029
Hypertension (%)	93.3%		90.7%		0.270
	**Mean**	**SD**	**Mean**	**SD**	***p***
Age	62.1	5.1	65.8	4.1	<0.001
BMI (kg/m²)	32.5	3.5	32.8	3.9	0.537
WC (cm)	112.7	9.5	104.3	9.6	<0.001
Systolic BP (mm Hg)	142.4	16.4	134.9	17.2	<0.001
Dyastolic BP (mm Hg)	88.4	10.2	83.8	9.6	<0.001
FPG (mg/dL)	101.0	20.3	102.4	26.8	0.611
Total cholesterol (mg/dL)	194	32	208	37	<0.001
HDL-C (mg/dL)	46	10	53	10	<0.001
LDL-C (mg/dL)	119	29	127	35	0.034
Triglicerydes (mg/dL)	165	77	168	72	0.798
HbA1c (%)	5.9	0.8	6.0	0.8	0.242
CV-ZS	1.0	2.5	−0.9	2.8	<0.001
Framingham score (%)	16.7	6.3	10.2	4.8	<0.001
Log ucOC (ng/mL)	1.4	0.8	1.6	0.8	0.043
MedDiet index	8.5	2.1	8.6	1.9	0.679

T2D: type 2 diabetes; WC: waist circumference; BMI: body mass index; BP: blood pressure; FPG: fasting plasma glucose; CV-ZS: cardiovascular risk Z-score; ucOC: undercarboxylated osteocalcin; MedDiet: Mediterranean diet.

**Table 2:** Evolution of MetS risk factors, CVS and serum ucOC levels.

	Baseline (n = 246)		6 Months (n = 227)		12 Months (n = 214)		
	Mean/ N	SD/ %	Mean/ N	SD/ %	Mean/ N	SD/ %	*p*
BMI (kg/m^2^)	32.4	3.6	31.6	3.9	31.3	4.1	<0.001
Waist (cm)	108.1	10.4	104.1	10.4	103.6	10.9	<0.001
Mean BP (mm Hg)	103.2	11.0	102.6	10.6	100.3	11.7	0.023
Pulse (bpm)	71.0	10.0	68.0	9.0	69.0	11.0	0.002
MedDiet index	8.5	2.0	9.9	2.8	9.8	3.1	<0.001
FPG (mg/dL)	102	24	99	24	99	24	0.105
HbA1c (%)	6.0	0.1	6.0	0.1	6.0	0.1	0.454
Total cholesterol (mg/dL)	201	36	205	39	200	38	0.201
HDL-C (mg/dL)	50	11	52	12	51	11	<0.001
LDL-C (mg/dL)	123	33	124	34	123	33	0.706
Triglycerides (mg/dL)	167	74	158	72	156	77	0.002
Log ucOC (ng/mL)	0.6	0.3	0.8	0.3	0.7	0.3	<0.001
CV-ZS	−0.2	0.2	−0.2	0.2	−0.2	0.2	0.956
Framingham index (%)	12.4	0.6	12.0	0.6	11.5	0.6	0.125
Sedentary	135	46	111	37	124	42	<0.001

BMI: body mass index; BP: blood pressure; bpm: beats per minute; FPG: fasting plasma glucose; MedDiet: Mediterranean diet; ucOC: undercarboxylated osteocalcin; CV-ZS: cardiovascular risk Z-score. ANOVA analysis of variance for multiple comparisons of means.

**Table 3:** Variables correlated with serum ucOC levels (log) at baseline, at 6 and 12 month follow-up.

	Baseline (n = 246)		6-Months (n = 227)		12-Monts (n = 214)	
MetS Patients	r	*p*	r	*p*	r	*p*
Age	0.065	0.312	0.122	0.065	0.141	0.039
FPG (mg/dL)	−0.102	0.110	−0.036	0.603	−0.177	0.013
HDL-C (mg/dL)	0.244	<0.001	0.147	0.036	0.155	0.038
LDL-C (mg/dL)	0.049	0.452	0.072	0.307	0.139	0.059
Triglycerides (mg/dL)	−0.113	0.079	−0.058	0.408	−0.021	0.773
HbA1c (%)	−0.193	0.007	−0.190	0.012	−0.265	0.002
Systolic BP (mm Hg)	0.017	0.788	0.041	0.558	0.072	0.319
Dyastolic BP (mm Hg)	0.012	0.848	0.071	0.304	0.179	0.013
CV-ZS score	−0.175	0.007	−0.054	0.457	−0.008	0.919
Framingham score (%)	−0.200	0.008	−0.068	0.390	−0.082	0.324

MetS: Metabolic syndrome; FPG: Fasting plasma glucose; BP: Blood pressure; CV-ZS: Cardiovascular Z-Score.

**Table 4:** Comparison of serum ucOC levels (log) in MetS patients with and free of prevalent T2D at baseline and at the 6- and 12-month follow-up, adjusting for sex and age.

Log ucOC (ng/mL)	MetS Patients (n = 234)			T2D Patients (n = 62)			*p*
	Mean	CI (95%)		Mean	CI (95%)		
Baseline (n = 246)	1.61	1.50	1.72	1.02	0.82	1.23	<0.001
6 months (n = 227)	1.99	1.88	2.10	1.57	1.34	1.80	0.002
12 months (n = 214)	1.70	1.60	1.81	1.14	0.93	1.34	<0.001

MetS: metabolic syndrome; T2D: type 2 diabetes; ucOC: undercarboxylated osteocalcin; CI: confidence interval.

**Table 5:** Relationship between ucOClevels (log) and CVR.

Sample	Time	B	CI		*p*
MetS patients	Baseline (n = 246)	−0.904	−1.955	0.148	0.092
	6 months (n = 227)	−0.148	−1.419	1.124	0.819
	12 months (n = 214)	−0.145	−1.427	1.137	0.824
MetS-No T2D patients	Baseline (n = 246)	−1.317	−2.417	−0.217	0.019
	6 months (n = 227)	0.014	−1.164	1.376	0.869
	12 months (n = 214)	1.014	−0.305	2.333	0.131

MetS: metabolic syndrome; T2D: type 2 diabetes CI: Confidence interval; T2D: Type 2 diabetes.

**Table 6:** MetS and T2D factors according to the 25th percentile of baseline serum levels of ucOC (log) in the total sample of MetS patients.

Log ucOC	Baseline					6 Months					12 Months				
	<0.92 ng/mL (n = 61)		≥0.92 ng/mL (n = 185)		*p*	<0.92 ng/mL (n = 25)		≥0.92 ng/mL (n = 202)		*p*	<0.92 ng/mL (n = 33)		≥0.92 ng/mL (n = 181)		*p*
	Mean	SD	Mean	SD		Mean	SD	Mean	SD		Mean	SD	Mean	SD	
BMI (kg/m^2^)	32.9	3.4	32.4	3.6	0.360	31.7	3.6	31.5	3.8	0.569	31.7	3.7	31.1	4.1	0.277
WC (cm)	109.7	9.6	107.2	10.2	0.312	105.6	9.8	103.6	10.2	0.464	106.1	10.8	102.7	10.7	0.143
Mean BP (mm Hg)	103.5	12.3	103.3	11.2	0.739	100.6	9.1	102.8	10.4	0.075	99.3	13.3	101.3	11.2	0.131
HDL-C (mg/dL)	45.1	7.7	50.4	10.3	0.002	47.2	8.9	53.5	12.2	<0.001	47.9	8.8	52.4	10.9	0.030
FPG (mg/dL)	110.1	33.6	99.0	20.7	0.002	106.2	34.0	96.4	19.9	0.009	111.5	33.8	95.1	19.4	<0.001
Triglycerides(mg/dL)	189.2	96.3	163.8	67.3	0.020	163.6	71.1	158.0	70.8	0.589	174.9	84.3	153.0	76.0	0.079
HbA1c (%)	6.3	1.0	5.8	0.7	<0.001	6.3	0.8	5.8	0.6	<0.001	6.4	0.9	5.8	0.6	<0.001
CV-ZS score	1.0	3.4	−0.3	2.4	<0.001	0.6	2.9	−0.3	2.5	0.035	0.7	3.0	−0.5	2.5	0.007
Framingham score (%)	15.1	6.7	12.6	6.1	0.107	13.7	6.7	12.1	6.6	0.386	14.1	7.0	11.8	6.5	0.413

BMI: body max index; WC: waist circumference; BP: blood pressure; FPG: fasting plasma glucose; CV-ZS: cardiovascular risk Z-score; ucOC: undercarboxylated osteocalcin.
